# The Book World of Medicine and Science

**Published:** 1898-01-08

**Authors:** 


### THE BOOK WORLD OF MEDICINE AND SCIENCE.

MASTOID ABSCESSES AND THEIR TREATMENT. By A. BROCA, M.D., Surgeon to the Paris Hospitals, and F. LUBET-BARDON, M.D., formerly *interne* to the Paris Hospitals. Translated and edited by HENRY J. CURTIS, B S., and M.D.Lond., F.R.C.S.Eng., Assistant in the Ear and Throat Department, University College Hospital. (London: H. K. Lewis. 1897. Price 6s.)

Dr. Curtis has done well in bringing this most useful book before English readers. The authors, one of whom is the son of the celebrated Broca, whose name will always be remembered in relation to the cerebral convolution which bears his name, have dealt very thoroughly with the subject which has given the title to their book, a subject which includes some of the most common sequences of suppurative middle-ear disease. Although it is through the tympanum that inflammatory mischief reaches the deeper parts, the tympanic cavity, to which so much attention is apt to be paid, is, in reality, of far less importance than the other petro-mastoid cells, and it is pointed out that probably a large part of the discharge in otorrhœa is produced in the mastoid cells, although it discharges through the tympanum. But if the opening by this route becomes occluded, then the products of inflammation can be no longer expelled; "the wolf will be locked up in the fold," and the symptoms characteristic of mastoiditis will be set up. Primarily, then, a mastoid abscess must be looked upon as a blind internal fistula, and if it is borne in mind that, once started, this is likely to go on until some exit is found, even while the original mischief in the tympanum may be progressing towards a cure, one sees how the treatment by operation falls into line with the general principles of surgery. It is quite clear, however, that in dealing with so complicated an organ many niceties of detail have to be considered, and to these this book forms a most admirable guide. Dr. Curtis has done his work as editor and translator extremely well. The clinical cases are interesting, and the coloured illustrations render the anatomical relations of the parts very clear. The work will be found a very useful manual, and will help to clear away a good deal of the mystery which has tended of late years to grow up around the subject.

A SYSTEM OF MEDICINE, BY MANY WRITERS. Edited by T. CLIFFORD ALLBUTT, M.A., M.D, F.R.C.P., F.R.S. Vol. IV. (London: Macmillan and Co. 1897. Price 25s. net.)

This volume commences with an announcement, for which all who have recognised the thoroughness of the work already done will be prepared, namely, that the System of Medicine will not be contained within the five volumes at first proposed. Professor Allbutt says that he did not at first realise "the increase of our knowledge from almost every point of view," and if one takes the issue of an authoritative "system" such as this, in which the opinions of leaders on every topic are condensed, as a form of stock-taking of current knowledge, and compares the outcome with what existed when a previous "system"—that of the late Sir J. Russell Reynolds—was published, one cannot fail to see the enormous advance that has taken place and the vast amount of new material which has been accumulated in recent years.

The volume now before us deals with diseases of the liver, of the kidneys, and of the lymphatic and ductless glands, and makes a commencement of the diseases of the respiratory organs. What one may call the intrinsic diseases of the liver are chiefly dealt with by Dr. William Hunter, who very properly prefaces his very difficult subject by a careful account of the functions of the liver and their disorders. The chapter on jaundice is a very able one; it sets before us the various theories that have been put forward, and after criticising them holds the balance in such a way as to give a fairly clear conception of the conditions which may lead to the production of toxæmic jaundice and of its clinical significance when met with. Obstructive jaundice is dealt with as it occurs among the various diseases of the liver. Dr. Andrew Davidson contributes a good article on suppurative hepatitis, which should be read along with one by Dr. Lafleur on amœbic abscess of the liver. The article on cirrhosis of the liver by Dr. Hawkins, and that by Dr. Hale White on perihepatitis must also be taken together and carefully compared. This is all the more necessary in view of the fact that very different views seem to be held by these authors as to the prognostic indication of ascites in cases of cirrhosis and as to the results of paracentesis in this disease. Dr. Mayo Robson's article on diseases of the gall bladder and bile ducts is an admirable essay on the subject. The article on diseases of the pancreas by Dr. Fitz is useful, showing that this organ is far from being so devoid of a pathology as some have imagined.

The section on diseases of the kidney commences by an article on the general pathology of the renal functions by Dr. Rose Bradford. The urine is first dealt with, and then many original observations are given regarding the physiology and pathology of the kidneys. The account of uræmia is full and interesting, but evidently the causation of this condition still remains a matter of considerable doubt. Into an essay of sixty pages Dr. Howship Dickinson has compressed a very complete description of those diseases of the kidney which are characterised by albuminaria, and this is followed by a series of articles by Mr. Henry Morris on those "other diseases" of the kidney in the treatment of which, perhaps more than in that of most disorders, the unity of medicine is made manifest, the physician having to know much of surgery and the surgeon much of medicine if either would give their patients the best advice.

Under the heading "Diseases of the Lymphatic and Ductless Glands" we find full and authoritative descriptions by Dr. Ord and Dr. Hector Mackenzie of the diseases of the thyroid gland, a subject in regard to which all that we can now call knowledge is entirely new since the days of the old "System of Medicine" of which this is the successor.

Dr. Rolleston deals very adequately and effectively with diseases of the spleen and with Addison's disease, and after a careful analysis of the various theories which have been propounded in regard to the latter, he brings it into line with diseases of other ductless glands, such as the thyroid, saying that it is due to an inadequate supply of supra-renal secretion. The description given of the symptoms of the disease is clear and good.

Professor Clifford Allbutt describes, under the name of scrofula, the various forms of adenitis which arise from irritation in the tonsils. Whether it is always due to the bacillus of tubercle he leaves open, but he quotes recent observations to show how frequently this may be the cause. Mr. Pridgin Teale follows with a good account of the surgical treatment of the disease, but it is to be noted that he speaks with some hesitation in regard to the very radical measures which have been adopted of late years for the removal of deeply situated glands. Sir Dyce Duckworth, in a careful article on obesity, very properly speaks of the absurdity of exalting the treatment of this condition "into a 'speciality'—a pretension unworthy of our profession and misleading to the general public." Diseases of the nose, pharynx, and larynx are ably treated by Sir F. Semon, Dr. de Havilland Hall, Dr. Greville MacDonald, and Dr. Watson Williams. The section devoted to these subjects forms a very complete treatise, which we are glad to see incorporated in a system of medicine. A general knowledge of all specialties should be possessed by all practitioners.

The volume altogether is one of great interest, and, although the individuality of the various authors is constantly made manifest, the editor has well maintained the proper balance between the various subjects.

